# Hidden struggles: professional Norwegian actors’ experiences with performance anxiety and its consequences in their daily work

**DOI:** 10.3389/fpsyg.2026.1803171

**Published:** 2026-07-10

**Authors:** Kristin Losvik Sørensen, Niklas Hald, Rose Mari Olsen, Kristin Berre Ørjasærter

**Affiliations:** 1 Faculty of Nursing and Health Science, Nord University, Mo i Rana, Norway; 2 Stockholm University of the Arts, Stockholm, Sweden; 3 Faculty of Nursing and Health Science, Nord University, Namsos, Norway; 4 Centre for Care Research Mid-Norway, Faculty of Nursing and Health Science, Namsos, Norway

**Keywords:** mental health, occupational health, performance anxiety, professional actors, qualitative study

## Abstract

**Introduction:**

Although performance anxiety is a well-known phenomenon among performing artists, little research has focused on professional actors. In this qualitative study, informed by a phenomenological hermeneutic and narrative approach, we explored how Norwegian professional actors describe and make sense of experiences commonly referred to as performance anxiety and how these experiences shape their professional practice.

**Methods:**

We conducted 14 in-depth interviews with professional actors using an interpretive, narrative-informed thematic approach.

**Results:**

Two themes were developed: ‘Big shoes to fill—Precarious professional legitimacy and escalating internalised standards’ and ‘Small sparks can cause big fires—Trigger events that destabilise perceived competence and precipitate spirals of anxiety’.

**Discussion:**

The findings suggest that performance-related anxiety is not merely an individual response to specific performance situations, but is shaped through ongoing processes of self-evaluation, professional identity, and contextual pressures within the acting profession. Experiences of anxiety were closely tied to internalised standards of performance, sensitivity to evaluative situations, and limited opportunities for openly addressing mental health struggles. A lack of openness between actors and their colleagues, especially directors and theatre managers, may further contribute to distress, illness, and early retirement among actors. This study offers an interpretive, context-sensitive understanding of performance related anxiety as socially embedded, highlighting the need to address both individual and contextual factors in supporting actors’ mental health.

## Introduction

Among performing artists, experiences often described as “performance anxiety” are widely recognised across performing arts as a major occupational challenge. According to the Australian Actors’ Wellbeing Study, up to a quarter of performers report having experienced debilitating performance anxiety during their careers (Maxwell et al., 2015). While such experiences are often discussed in terms of symptoms and prevalence, less attention has been paid to how they are understood, interpreted, and made meaningful by performers themselves within their professional contexts. In this article, we explore how professional Norwegian actors describe and make sense of experiences commonly referred to as performance anxiety in different professional settings, including on stage and on film sets, and the consequences of those experiences.

Although mild stress and anxiety before or during a performance can improve an artist’s performance (Fernholz et al., 2019, p. 32; Nicholl and Abbott, 2024; Racine et al., 2025), performance anxiety that reaches an inhibiting and/or debilitating level can reduce well-being and increase stress and anxiety (McDuff et al., 2025). At its worst, performance anxiety can result in prolonged sick leave (Meyer-Dinkgräfe et al., 2012), early retirement from performing (Fernholz et al., 2019), or harmful coping strategies such as using alcohol and/or tranquillisers (Studer et al., 2011).

Manifestations of what is commonly referred to as performance anxiety are often divided into four categories: physiological symptoms, emotional conditions, cognitive conditions, and behavioral changes that can impair professional performance (Hague, 2016; Meyer-Dinkgräfe et al., 2012). For one, physiological symptoms include increased heart rate, sweating, shortness of breath, shaking, numbness in the fingers, clammy hands, dry mouth, upset stomach, dizziness, and difficulties with speaking (Fernholz et al., 2019; Hague, 2016; Meyer-Dinkgräfe et al., 2012; Niering et al., 2023; Simmonds and Southcott, 2012). For another, emotional conditions include a fear of failure, dissatisfaction with one’s performance (Meyer-Dinkgräfe et al., 2012), panic, and stress (Fernholz et al., 2019), while cognitive conditions include a loss of confidence, lack of concentration due to interfering thoughts about performing, memory lapses, and interference in the creative process (Fernholz et al., 2019; Meyer-Dinkgräfe et al., 2012; Steptoe et al., 1995). Last, behavioral changes include social avoidance, unsafe behavior [e.g., alcohol abuse; (Fernholz et al., 2019; Meyer-Dinkgräfe et al., 2012), and eating difficulties (Steptoe et al., 1995)].

Likewise, various approaches to understanding the manifestations of performance anxiety exist as well. Theory on music performance anxiety (Kenny, 2011) has extended the perspective from examining direct encounters with an audience—that is, stage fright (Koninj, 1991; Lemasson et al., 2018)—to situations in which artists are required to perform, including auditions and rehearsals (Fehm and Schmidt, 2006; Fernholz et al., 2019). Meanwhile, in terms of duration, performance anxiety can creep up weeks or months before performing (Studer et al., 2011), first while visualising and planning the performance (Gabbard, 1981; Kaplan, 1969; Simmonds and Southcott, 2012), and continue after the performance in the form of a fear of reactions and feedback (Lehrer et al., 1990). Beyond that, it can even influence how a performer envisions their future career (Kenny, 2011; Steptoe et al., 1995).

Although performance anxiety is one of the most-described phenomena among performing artists, few studies have focused on the phenomenon among professional actors in particular (Goodman and Kaufman, 2014; Kumar, 2019). Instead, research on performance anxiety among professional performers has focused on musicians (Fernholz et al., 2019; Nicholl and Abbott, 2024; Racine et al., 2025) and, to a lesser extent, dancers (Fernholz et al., 2019; Goodman and Kaufman, 2014; Nicholl and Abbott, 2024; Racine et al., 2025; Valentine et al., 2006). To date, research on actors has chiefly examined stage actors, with particular focus on stage fright and how the phenomenon occurs or is experienced (Brennan, 2011; Goodman, 2012) and how it might be treated (Meyer-Dinkgräfe et al., 2012). Despite a doctoral thesis on former child actors’ mental health (Breslin, 2025), to our knowledge no studies have addressed performance anxiety on film or TV sets. Even so, environmental factors such as job insecurity, power imbalance, a culture of not talking about psychological struggles, and a fear of one’s reputation are known to impact actors’ mental health and well-being (Robb et al., 2018), as research on Norwegian actors’ experiences with their occupation has recently revealed (Sørensen et al., 2024).

Rather than treating performance anxiety as a fixed or predefined construct, this study approaches it as a range of experiences that actors themselves interpret and negotiate in relation to their professional practice. Thus, more research on actors’ experiences with performance anxiety and a broader approach to the phenomenon, including a pre- and post-performance perspective, is desired in the field (Kumar, 2019). In response, the aim of our study was to investigate how professional actors describe and make sense of their experiences commonly referred to as performance anxiety across professional contexts (e.g., stage, auditions, rehearsals, and film and TV sets) and how it affects their professional functioning and mental health.

Drawing on a phenomenological hermeneutical and narrative-informed approach, we explore how professional actors describe and make sense of situations involving insecurity, fear, and perceived performance pressure.

To guide our exploration, we devised two research questions (RQs):

RQ1: How do professional actors describe and make sense of experiences of anxiety and pressure across work contexts?

RQ2: What perceived consequences do these experiences have for their professional functioning and mental health?

By attending closely to actors’ own narratives, this study contributes to literature by offering an interpretive and context-sensitive understanding of performance-related anxiety, highlighting how such experiences are shaped not only by individual factors, but also by professional expectations and cultural contexts within the acting field.

## Materials and methods

Our qualitative study involved in-depth interviews with professional actors and an exploration of their narratives describing their experiences commonly referred to as performance anxiety in their day-to-day work. We drew on data from a broader qualitative phenomenological hermeneutic study concerning professional Norwegian actors’ experiences of anxiety, pressure, and professional insecurity in performance related contexts, and how these experiences affect their mental health.

To enhance reflexivity throughout our research process, we continuously reflected on how our disciplinary backgrounds and assumptions might have shaped our interpretation of the data and our analytic decisions. Such reflexive work was supported by ongoing dialogues with two professional actors and a psychologist who served as expert consultants. The actors offered insights from diverse experiences in Norway’s entertainment industry, while the psychologist contributed valuable insights from his research on musicians’ mental health in the same industry. Together, their practical and theoretical knowledge about acting and performers’ psychology boosted the study’s validity and provided a basis for important dialogues during data analysis and while discussing the findings.

### Participants

Our study’s participants all worked as professional actors for more than 3 years in the community of freelance actors and/or in various theatre institutions. Some also had experience with film and TV productions.

To recruit participants with relevant experience and ensure the potential for rich narratives, we targeted professional networks and stipulated voluntary participation. Most participants were recruited in response to advertising on the online platforms of the Norwegian Actors’ Association, the Norwegian Actors’ Centre, the ‘Alliance for Actors and Dancers’ and ‘Drama and Theatre Educators’ or via social media. The first author also contacted theatres across Norway by mail to raise awareness about the study and encourage actors to participate.

The participants’ experiences represented not only a large swath of Norway but also an array of performance settings, including independent theatre groups, musical productions, and national and regional theatres. Four participants also possessed some or extensive experience in film and TV, while three others had stopped acting professionally and taken positions in other professions in and outside the entertainment industry. Table 1 presents the demographic characteristics of the sample.

**Table 1:** Demographic characteristics of the sample (*N* = 14).

Demographic characteristics of the sample (*N* = 14)		
Variable	Category	*n*
Gender	Woman	9
	Man	5
Age, in years	20–29	5
	30–39	4
	40–49	4
	50–59	1
Years of professional experience	Range	<5–20+
	Mean (estimated)^a^	13.2
Work context^b^	Theatre	14
	Film and/or TV	4
Current work status	Long-term employed	3
	Freelance	8
	Other	3

a Mean years of experience was estimated based on interval midpoints.

b Participants could report more than one work context.

### Data collection

Data were collected in 2022–2023 during 14 in-depth interviews, each lasting approximately 90 min. One interview was conducted by phone, whereas the other 13 were conducted virtually on Zoom. In narrative research using in-depth interviews, 6–10 participants are considered a large enough number to obtain rich data (Dahal et al., 2024). The interview approach did not focus on predefined concept such as performance anxiety. Instead, participants were invited to describe their everyday work as actors. The interview consisted of open-ended questions (e.g., ‘Would you describe a typical day in your work as an actor?’; ‘Would you describe a really good day and a tough day in your job as an actor?’; and ‘What is meaningful to you about being an actor?’) to encourage participants to freely express their experiences (Riessman, 2008). Descriptions of anxiety, pressure, and insecurity emerged as part of these narratives and were interpreted in relation to how participants themselves made sense of such experiences.

Afterwards, the first author transcribed the audio-recordings of the interviews, and any identifying information (e.g., named events and productions) was changed or removed to protect the participants’ anonymity. The interview guide is presented in Appendix 1.

Most participants were freelancers. As the interview process progressed, the data became increasingly rich and internally coherent, with participants articulating overlapping experiences and interpretations. In phenomenological research, sample size is less important than the richness and depth of the data obtained. This highlighted the need to include broader perspectives, such as those of permanent staff and more experience actors in film and television. To address that oversight, the first author reached out to several theatre institutions and participated in a podcast hosted by a famous Norwegian actress, Verndal (2022), to raise awareness about the study and encourage actors from the mentioned groups to contact her. As a result, we were able to recruit four additional participants, three of whom were long-term employees and two of whom had significant professional experience in film and television production.

### Data analysis

Data analysis focused on how participants constructed and made sense of their experiences across different professional contexts. We adopted an interpretive, narrative-informed thematic approach inspired by Riessman’s (1993) narrative analysis and Braun and Clarke’s (2022) reflexive thematic analysis.

The analysis involved iterative movement between individual narratives and patterns across cases. From Riessman (1993) we followed a three-phase process of telling, transcribing, and analysing. In the telling phase, participants shared rich narratives about their workdays. To thoroughly comprehend each participant’s situation, we provided participants with considerable room for full descriptions of their backgrounds. The telling phase also gave the investigator—in our case, the first author—useful information about how each story was told. Along those lines, the first author gave a video-recorded reflection of each interview immediately after it was conducted. Next, in the transcribing phase, the first author transcribed all interviews verbatim, which afforded a firm grasp on the written text and the ambiguities in the language and thus provided valuable insights into the meanings of the stories told. The first author had a second online meeting with the participants to ensure that the material was sufficiently de-identified, which also afforded valuable insight into initial impressions of the narratives from the participants’ perspective. Most of the participants granted permission to share anonymised transcription with the other co-authors. The first author also held regular meetings with the co-authors and the expert group.

In general, narrative analysis focuses on the content of the participant’s narrative and less on how their story is told, the structure of speech selected, or the context of the conversation (Riessman, 2008). Even so, subsequent impressions from the telling and transcribing phases provided valuable background information for the analytical process. The analysis began inductively by having the first author read the transcripts thoroughly many times to obtain a rich picture of the participants’ narratives. The video-reflected recordings of the first impressions were useful in that part of the analysis in order to remember the settings of the interviews and the first author’s impression of the participants. Three episodes in which participants described signs of panic attacks or instances of stage fright in professional situations were selected for in-depth analysis. Narrative meaning units were sorted out from the stories, including ‘He felt he did not “fit in” among the other famous actors’ and ‘A tiny mistake in one of her strengths, “remembering lines,” makes her lose her confidence’, and discussed with the expert group and the co-authors. In the third step of the analysis, important meaning units were identified across the narratives, and three cross-cutting themes were articulated, namely ‘No one wants to hire a mad actor’, ‘The bug’, and ‘The great pretender’. Through written and oral discussions among the authors during the development of the performance anxiety definition, the themes were narrowed down to two: ‘Big shoes to fill’, and ‘Small sparks can cause big fires’. An example of the narrative analysis is presented in Appendix 2.

To explore the material utterly and include relevant accounts that had been omitted due to not containing episodes similar to panic attacks, a reflective thematic analysis was conducted of the data. The thematic analysis involved examining the full dataset, with particular focus on the participants’ experiences of performance anxiety. Relevant meaning units were coded. The analysis process ended by grouping the codes into subthemes and themes (Braun and Clarke, 2022). The result was a revision of the two cross-cutting themes: *‘*Big shoes to fill – Precarious professional legitimacy and escalating internalised standards’, with the subthemes ‘Fear of others’ judgement’, ‘Getting the job “by accident’, and ‘Not deserving the job’; and Theme 2, ‘Small sparks can cause big fires—Trigger events that destabilise perceived competence and precipitate spirals of anxiety’, with the subthemes ‘The lack of feedback’, ‘Never good enough’, and ‘Small events—with big impacts’. The analysis of Theme 1 and Theme 2 is presented in Appendix 3 (see Table 2).

**Table 2:** Example of Theme 1.

Example of Theme 1		
Meaning unit	Subtheme	Theme
I’ve felt it before … that people around me say, ‘Isn’t he going to quit soon?’ or ‘Isn’t it time he looked in the mirror and saw how bad he is?’ Well, that doesn’t quite match the reactions I’ve gotten to things I’ve done, but still, there’s this nagging feeling that I’m not good enough … that I myself should at least make the choice to start something else. (Kristian)	Fear of others’ judgement	Big shoes to fill—Precarious professional legitimacy and escalating internalised standards
You’re terrified that others will see it. That other actors will think, ‘Oh, he’s lost it’. (Jens)		
Yeah, my nerves are getting worse and worse. I reckon it’s down to age. I thought maybe some people become more like, ‘Nah, I don’t give a damn what people think of me’ as they get older. … But I don’t feel it’s been quite like that for me. I feel it’s perhaps been a bit the other way round, that you become a bit more self-conscious about things. (Katrine)		

Some meaning units have been excluded from the table to protect the participants’ anonymity.

The first author was responsible for the initial draft of the manuscript, supported by co-authors’ written and oral feedback across all stages of analysis and writing. Findings from both analysis processes have been used in the results section.

### Ethics approval

The Norwegian Agency for Shared Services in Education and Research approved our study’s personalised data handling approach based on national legislation (No. 956883/2022). The study was also subjected to a pre-assessment by the Regional Committees for Medical and Health Research Ethics, which concluded that our study was not a medical or health research project falling within the scope of the Health Research Act.

Informed written and oral consent was obtained from all participants before participation. During the research process, participants were informed orally and in writing about their right to withdraw their consent to participate at any time for any or no reason.

To ensure anonymity, we have assigned pseudonyms to all participants quoted in this article. Due to the small size and visibility of Norway’s entertainment industry, each participant reviewed their anonymised transcript together with the first author to ensure that their anonymity was maintained.

## Results

In our study, we gathered 14 professional actors’ descriptions of and reflections on experiences commonly referred to as performance anxiety in their day-to-day work and analysed how they made sense of these experiences across different professional context. Rather than treating performance anxiety as a predefined category, the analysis focused on how such experiences were narrated, interpreted, and embedded in actors professional lives. To capture the breadth of their experiences from a narrative perspective, we have given more space to Jens’ and Jannes’ stories from the stage and a film set. The analysis revealed two themes: ‘Big shoes to fill—Precarious legitimacy and escalating internalised standards’ and ‘Small sparks can cause big fires—Trigger events that destabilise perceived competence and precipitate spirals of anxiety’.

### Theme 1: big shoes to fill—precarious professional legitimacy and escalating internalised standards

Theme 1 describes the psychological burden of high expectations in performance-intensive contexts. More specifically, it captures how actors’ sense of professional legitimacy is experience as fragile and continuously negotiated, rather than stable or secured. Our analysis revealed an ambivalence about legitimacy, particularly between its acknowledgement and doubts about it. Being offered an acting job creates strong feelings of happiness and humility in being allowed to work in acting. At the same time, those moments are accompanied by insecurity about one’s talent and an eagerness to prove one’s worthiness for the job. This ambivalence can be understood as a tension between external validation and internalised self-doubt, where recognition does not resolve insecurity but instead intensifies self-scrutiny (Robb et al., 2018). Such ambivalence manifested when participants described working in high-profile productions or feeling ‘lucky’ to have been casted. Participants stated that years of experience in the business did not necessarily reduce those feelings; on the contrary, a persistent, nagging sense of insecurity and low self-efficacy were described as accompanying them throughout their careers and, in some cases, contributing to reflections about leaving the profession.

Nina described how, after landing one of her very first parts as an actress, she struggled with an inner conflict shaped by perfectionism and professional identity about whether she deserved the job. The feeling overshadowed the joy of having succeeded, landing a role, and, above all, doing what she loves as a professional:

I remember we did one of those promo videos for a musical that was going to be posted on YouTube. And when it’s finished, you can just see how my body is like, ‘Oh sorry, I’m just going to go’. I mean, I apologised a lot for being there. [*Pause*]. And I was completely overwhelmed by being allowed to work on something I loved and a bit unsure whether I actually had the right to do it. (Nina)

Nina’s account can be understood as reflecting a fragile sense of professional legitimacy, which appears comparable to impostor phenomenon (Clance and Imes, 1978). Where being selected does not confirm competence but instead triggers doubts about deservingness. Rather than reinforcing confidence, the experience appears to be internalised through a lens of self-criticism, suggesting that success may heighten rather than alleviate insecurity (Sims and Ryan, 2024).

Similarly, Kristian, one of the oldest participants, described the pressure as a constant fear that others considered him to be untalented as an actor, which only intensified after he left a theatre institution and began working as a freelance actor with, in his words, ‘various successes’. Despite positive feedback from the audience, his nagging fear of other actors’ sarcastic comments led him to quit acting before he became a laughingstock in the acting community. This combination of self-monitoring and anticipatory anxiety may have contributed to making his worries self-fulfilling. Kristian never knew whether other actors indeed regarded him in that way, but the fear of it grew so strong that he ultimately pursued a different occupation:

The feeling I have, in a way, is probably something similar to the Law of Jante [from Aksel Sandemose’s novel *A Refugee Crosses His Tracks* (1933)], but I’ve felt it before … that people around me say, ‘Isn’t he going to quit soon?’ or ‘Isn’t it time he looked in the mirror and saw how bad he is?’ Well, that doesn’t quite match the reactions I’ve gotten to things I’ve done, but still, there’s this nagging feeling that I’m not good enough … that I myself should at least make the choice to start something else. (Kristian)

This account can be understood as a form of anticipatory self-monitoring, where imagined evaluations from others shape self-perception and decision-making (Gütges et al., 2025). His reference to the Law of Jante can further be interpreted as a cultural frame that legitimises self-doubt, positioning feelings of inadequacy as socially grounded rather than purely personal. In this way, self-criticism is reinforced through shared cultural narratives, making it more difficult to challenge or reframe (McLean et al., 2023).

Last, we turn to Jens, a young actor whose experience illustrates how performance anxiety in interaction with professional identity can escalate under high-stakes conditions. Jens had never had any experiences with poor mental health, despite some episodes of hyperventilation as an acting student. After a year of freelance acting and work in various other occupations, Jens was unexpectedly offered what he called ‘a chance of a lifetime’ to perform on a major stage. The opportunity arose when a theatre in another town urgently needed a replacement, and Jens’ name came up. Because the director had seen him in a small part elsewhere, Jens got the job:

I was only in my early 20s, and looking back, I’ve realised it was a bit early to get into it. It would have been more natural for me to work at a smaller theatre first, perhaps, but I ended up straight into a big theatre. [It was] a brilliant opportunity, but it meant that you were constantly—you’d bump into, like, big-name actors in the canteen every day, and it all just became a bit absurd, sort of. On performance number twenty, I suddenly started having trouble standing still on stage. Often in the theatre world—at least in the musical world—you suddenly ‘freeze’; you ‘freeze’ right in the middle of a scene, whilst something important is happening in some spotlight, you know. And there I suddenly started getting this really bad shaking just from standing still. (Jens)

Jens’ experiences can be understood as a shift from automatic to controlled performance, where increased self-awareness disrupts previously embodied competence. Bodily sensations such as shaking are interpreted as signs of failure, suggesting a process in which attention turns inward and amplifies the experiences of losing control (Sokoli et al., 2022). Rather than being a discrete event, this episode illustrates how pressure, visibility, and perceived expectations interact to escalate anxiety over time.

### Theme 2: small sparks can cause big fires—trigger events that destabilise perceived competence and precipitate spirals of anxiety

Theme 2 captures how initially small, seemingly insignificant events, negative comments, or the lack of feedback can grow into potentially career-changing pitfalls and escalating experiences of anxiety. More specifically, it highlights how actors interpret small disruptions as threats to competence setting off cascading process of self-doubt (Taylor, 2016).

Participants described how uncertainty about and failure in what they considered to be their strengths was akin to pouring gasoline on the fire and only compounded the negative consequences of their anxiety. Sometimes when they observed more experienced actors fail, they became aware of the threat, followed by symptoms such as shaking, chest pains, and a fear of failure. This pattern can be understood as a heightened sensitivity to evaluative cues, where minor incidents are interpreted as indicators of broader inadequacy (Pietrzak et al., 2005). Due to organisational silence, sharing such feelings was difficult. Participants expressed concerns about worrying the people in charge or getting a bad reputation within their professional community. At the same time, the participants also highlighted the importance of putting those feelings into words and how a verbal manifestation of the symptoms seemed to mark a turning point for their health. This suggests that anxiety is not only internally generated but also maintained through social and organisational conditions that limit opportunities for disclosure and reassurance (Robb et al., 2018).

Nina, for instance, described how her creative qualities as an actor succumbed to fear based on constant negative comments from her teachers that had effectively inhibited her. The teaching method broke the students down but did not focus on re-strengthening them afterwards:

Even after leaving [acting school], I still hadn’t managed to learn how to use my creativity. I remember so clearly the last 6 months [at acting school] when one director was just like, ‘You have to improvise! Improvise!’ And no one dared to improvise anymore because, well, every time we tried something, it was wrong. (Nina)

This account illustrates how experiences of repeated negative evaluation may become internalised, shaping later inhibition and reduced creative risk-taking. Rather than fostering development, such environments appear to contribute to lasting patters of self-doubt (Innes, 2021).

The lack of explanation about creative decision-making in theatrical productions was also a source of increased performance anxiety during rehearsals. Another actress, Veronica, described an episode in which one of her solo songs in a show had been removed without explanation, how it destabilised her, and contributed to cognitive patterns associated with performance anxiety, such as perfectionism. That made her not able to remember any material, old or new, the rest of the day:

I had spent the whole weekend learning the new songs, and one of them was a solo song. Then I arrived on Monday, and the first thing I know is that the solo song has been cancelled. … And so, well, I couldn’t do it; I couldn’t do the songs I’ve practised. … Everything was in place for me to perform, and then I couldn’t do it. … So, when the day ended … I noticed that the new songs that we learned that day didn’t stick, because my head had started thinking, ‘Why did they cut it [the solo song]?’—because I can’t sing’. (Veronica)

Veronica experience can be understood as an example of how abrupt changes and lack of explanation destabilize perceived competence (Innes, 2021). The absence of feedback creates space for self-critical interpretations, which in turn interfere with cognitive functioning, such as memory and concentration.

Janne, a prominent actress in Norway with extensive experience in acting, had endured a challenging job assignment that took a significant mental toll on her. Without the time to decompress from the experience, she simply moved on to the next project. A tiny incident on set gave her a major physiological, emotional and cognitive response associated with performance anxiety:

It all started when a colleague suddenly completely blanked out during filming. You saw this high-profile person just completely fall apart. And she had to be fed line by line by the director, and people were shaking their heads, and time was ticking away, and it was a total crisis. And then suddenly, I started thinking, ‘Oh, what if that had happened to me?’ And I made a tiny mistake when I said my line, and then that thought started spinning, until I managed to twist myself into a kind of performance anxiety hell. My heart rate was, I mean, it was going so fast, because I was in a complete panic. And I wanted to hide this from everyone, because I’m known for being bloody good with lines.

Janne’s account illustrates how observing others’ failure can activate anticipatory anxiety, where imagined future scenarios trigger immediate physiological and cognitive responses (Askew and Field, 2008).

The turning point of her story was to tell her mum, one of the other actresses on set, and, at last, a psychologist:

I told Mum [*laughs*]. And that helped a bit. And then I spoke to [an actor]. I just had to tell someone about it on set. I didn’t want the director to get worried, or for it to become a ‘thing’ or something like that. Also with the psychologist saying, ‘You’ll get through it’.

Her decision to share her experience can be interpreted as a critical turning point, suggesting that verbalising distress and social support may interrupt escalating anxiety (Sun et al., 2026). At the same time, her hesitation to inform the theatre director reflect broader professional norms discouraging openness about vulnerability (Sørensen et al., 2024).

## Discussion

Building on the two themes developed in the analysis—‘Big shoes to fill – Precarious professional legitimacy and escalating internalised standards’ and ‘Small sparks can cause big fire—Trigger events that destabilise perceived competence and precipitate spirals of anxiety’—this discussion deepens the understanding of how actors describe and make sense of performance-related anxiety in their everyday professional lives. Rather than being confined to discrete performance situations, our results suggest that such experiences are embedded in ongoing processes of self-evaluation, identity formation, and professional legitimacy within the acting field.

Given the dominance of freelance theatre actors in the sample, our findings suggest that sustained performance pressure, increased self-criticism over time, and a reluctance to disclose mental health struggles may, within this group, shape how experiences of anxiety and pressure are interpreted and managed, and how they may contribute to worsening difficulties over time. They also illuminate that limited openness within theatre environments, particularly in relationships with directors and managers, may have consequences for actors’ health and long-term participation in the profession. The precarious nature of the profession leaves freelance actors more exposed than permanently employed actors.

Most broadly, the findings demonstrate how actors experience and interpret anxiety related challenges and sustained performance pressure across all stages of their careers. For some, these experiences begin not only in performance situation, but earlier during their acting education. Although pressure with performing is often embraced as a force for development, our findings also suggest that it may also be internalised as ongoing self-monitoring and self-evaluation contributing to emotional and cognitive strain. Despite describing significant strain, participants rarely reported behavioral withdrawal, such as avoiding uncomfortable situations. This pattern can be understood as reflecting how professional norms and perceived replaceability, shape actors’ responses, making persistence not only personal choice but also a socially conditioned necessity. Considering that many actors perceive themselves as easily replaceable (Hald, 2022), such findings reveal not only determination but also a fear of reputational consequences lined to stepping back or disclosing difficulties. Those findings reflect Sørensen et al.’s (2024) observations that ‘quitting’ is not a word in the actor’s vocabulary, that they rarely take sick leave, and that they always finish the job. Such pressure seemed to keep our participants going until they became ill or decided to leave the profession for good. Prolonged exposure to work-related stress generally increases the possibility of burnout, especially when combined with performance anxiety (Stocking, 2016), and that risk raises concerns about the long-term health implications of the work culture of actors. Although understudies are common in large-scale shows and musicals (Hald, 2022), they are rare in regular theatre and film, which gives actors limited scope for recourse should they need a break.

Our findings may suggest that self-criticism and high levels of perfectionism can develop into symptoms of severe performance anxiety. Similar patterns have been noted in previous research, wherein acting students have reported feeling exposed and engaging in inner dialogues that frame their performances as inadequate (Robb and Due, 2017) and wherein professional actors have described comparable self-critical monologues (Brott et al., 2025). Our findings suggest a process of how negative experiences, including perceived failure, can exacerbate performance anxiety even after many years in the entertainment industry. A recent Australian study has also confirmed the prevalence of self-criticism and doubts about one’s own acting methods (Brott et al., 2025). Along those lines, Goodman’s (2012) research on elite actors and stage fright, which is considered as a debilitating version of performance anxiety (Powell, 2004), has identified a lack of confidence as an important source of stage fright. In response, Clements (2022) has recommended mentoring programmes for newly educated artists in the entertainment industry. However, if environmental factors continue to foster negative self-appraisal, access to guidance and professional follow-up may be important throughout their professional lives and not just in the beginning of their careers.

Our findings point to the possibility that both negative feedback and the absence of feedback from teachers and directors can strongly affect actors. Innes (2021) research on preventing performance anxiety among student actors has highlighted the importance of avoiding teaching practices that ignore or abuse the power dynamic within elite training institutions to increase the students’ resilience. At the same time, our findings suggest that a lack of feedback can foster professional insecurity and intensify self-criticism, both of which may contribute to escalating spirals of anxiety.

A recurring theme in our findings suggests that actors believe personal issues should not interfere with a production’s progress, especially when the challenges involve mental health. Previous research has similarly shown how a culture of ‘leaving your personal problems at the door’ (Robb et al., 2018) and notes that future job opportunities are often controlled by artistic leaders (Sørensen et al., 2024), which further discourages disclosure. Lubert et al.’s (2025) research project on ways to prevent debilitating performance anxiety among performers has emphasised the need for good artist–coach relationships and the need to work with artists in their unique contexts. This gives actors in long-term contracts an advantage because they have time to develop these relationships (Sørensen et al., 2024). Openly disclosing physical or mental health struggles is often perceived as inviting judgment, threatening professional credibility, and potentially interfering with an actor’s career.

Overall, our findings raise the possibility that the actors in our study did not express specific experiences of mental health difficulties or debilitating performance anxiety early in their careers. By contrast, research on music performance anxiety in musicians has shown that such anxiety may overlap with other mental health issues (Kenny, 2011) and sometimes can be observed from a very early age (Kenny, 2026). Given the small sample of participants in our study, the apparent difference between musicians and actors warrants further investigation.

A paradox emerging from our findings suggests that, for actors, remaining silent about how they feel and not disclosing it to anyone creates long-term problems, often intensifies symptoms of performance anxiety, and ultimately impairs both their well-being and acting performance. Among our participants, even minor symptoms such as shaking could generate fears of not being taken seriously and further dissuaded them from seeking help with their struggles. Being a freelance actor seemed to raise the threshold for seeking help and justified ignoring the body’s signals that something was wrong. After all, a bad reputation can spread fast in Norway’s small entertainment business (Sørensen et al., 2024). If an actor’s problems became too severe for them to handle independently, then they typically turned to their private network and/or trusted colleagues for help. That observed dynamic is consistent with Szabó et al.’s (2022) results from Australia indicating that actors often turn to personal contacts instead of institutional resources. Ironically, while concealment is intended to protect their careers, it may obstruct an essential first step towards recovery: telling someone. Research on stage fright among children shows that systematic and structured approaches to identifying and classifying the severity of stage fright can facilitate early support and the development of coping strategies for individuals at risk (Mastrothanasis et al., 2023).

### Methodological strengths and limitations

As authors, our diverse backgrounds contributed valuable insights into the field but also introduced various potential biases. To begin, we brought a wealth of expertise in nursing, acting, and mental health research. The first author is a registered nurse with experience in detoxing and rehabilitation within specialist care and holds a bachelor’s degree in applied drama and a master’s degree in professional science. The second author has extensive experience in acting, holds a doctoral degree in professional science, and has served as a lecturer in a university department dedicated to acting in Sweden. The third author is also a registered nurse with leadership responsibilities at a care research centre and holds a professorial position at a university in Norway. Last, the fourth author is a trained clinical social worker with 15 years of experience at psychiatric outpatient and inpatient clinics within specialised mental health services and possesses research expertise in mental health, the arts, and well-being. However, our experiences might also create a biased view of the material and prevent us from seeing it in a new light. On that count, the discussions within the research team and with the expert group during the analysis were an important, valuable counterbalance.

Meanwhile, regarding our study’s limitations, the participants were recruited by self-selection. Self-selection as a recruitment strategy has a possibility of self-created bias based on the participants’ individual needs and characteristics (Kaźmierczak et al., 2023). Consequently, the findings might not be fully generalisable to the wider population of actors in Norway (Novosel, 2023). A more heterogeneous sample, including one with a greater proportion of actors with film and/or TV experience, as well as permanently employed actors, might have given richer, more nuanced data material.

Along the same lines, the small group of participants might not have captured the full range of performance anxiety among professional actors, but it offers important starting points for future in-depth research. Freelancers, primarily with experience in theatre acting, constitutes the largest subgroup of participants, which likely influenced the findings and limits their transferability to permanently employed actors and those working mainly in film and television. While film and TV actors work in a project-based context as freelancers, we did not explore in depth the experiences of permanently employed actors in institutional theatres, nor how organisational conditions shape their performance anxiety. The findings suggest that close relationships with directors or theatre managers may be important for fostering open dialogue about performance anxiety, which highlights the need for future research to examine how employment structures and leadership practices influence help-seeking and disclosure.

## Conclusion

Few studies have examined how actors describe and experience performance anxiety and its consequences for their professional practice (Goodman and Kaufman, 2014; Kumar, 2019; Meyer-Dinkgräfe et al., 2012). Our study’s findings suggest that the constant internal and external pressure to perform can be highly demanding and might have serious consequences for actors’ health. A key finding of our study which included a majority of freelance actors, is that the actors claim not to have any significant prior experience with mental health issues or severe stage fright. For some, symptoms of performance anxiety seem to increase with experience and time spent in the entertainment industry, thereby suggesting that age and seniority are not automatically protective factors against those experiences.

Actors’ refusal to share their struggles may also exacerbate anxiety and negatively affect stage performance. The lack of openness between actors and their colleagues, especially directors and theatre managers, may additionally contribute to illness and early retirement from the business. Another barrier to open dialogue is the perception that sharing one’s problems is pointless because solutions are unattainable.

### Implications for practice and research

Our findings highlight the need for a support system and increased awareness about performance anxiety among actors. Developing targeted programmes and/or resources may help actors to recognise early signs of distress and equip them with strategies to manage challenges concerning mental health.

Further investigations on how early intervention as well as actors’ knowledge of early signs of performance anxiety affect mental health and well-being are therefore of interest. Actors’ limited understandings about early signs of performance anxiety also highlight the need to strengthen targeted training in emotional regulation, stress identification, and self-care strategies in actor education programmes. Such training would better equip actors to recognise and respond to emerging mental health challenges.

Our findings also show that early symptoms of performance anxiety can seem insignificant and are easily overlooked until they develop into conditions that are nearly impossible to manage. Combined with a lack of knowledge about the signs of performance anxiety, increases the difficulties of taking the signs seriously.

Our results also reveal that even seemingly insignificant incidents could have a big impact if they clash with an actor’s self-image. The nomadic lives of freelance actors and their fear of seeming difficult if they complain (The Big Freelancer Report, 2021) underscore even more strongly that productions need to have solutions or meeting points outside the rehearsal room in order to build trusting ensembles and be able to detect when someone on the team is struggling.

Last, research on how actors’ mental health may be affected by different ways of organising the entertainment business—for example, by using full-time employees and freelance actors or by having understudies in regular theatrical performances—could make valuable contributions to studies on actors and their mental health and well-being.

## Data Availability

The datasets presented in this article are not readily available because the participants did not consent to making their interview transcripts publicly available, for the transcripts contain personally identifying information. Thus, for ethical and security reasons, the interview transcripts and the study’s internal administrative documents are not publicly available. Requests for those materials can be sent to the corresponding author. Requests to access the datasets should be directed to Kristin Losvik Sørensen, kristin.sorensen@nord.no.
