# Supplementary material for: Hidden struggles: professional Norwegian actors’ experiences with performance anxiety and its consequences in their daily work
Source: Front Psychol. 2026 Jul 10;17:1803171. doi: 10.3389/fpsyg.2026.1803171 (PMC13397583; doi:10.3389/fpsyg.2026.1803171)
Supplement: Supplementary file 1 [file Data_Sheet_1.PDF]

Appendix 1

Interview guide

Topic 1: Background

- **Can you tell me a little about yourself?**
    - *General details*
    - *Family, children, marital status, etc.*
    - *What made you want to become an actor?*
    - *What qualifications do you have?*

Topic 2: What is it like to work as an actor?

- **Can you describe a typical working day as an actor?**
    - *What are rehearsals, auditions, working conditions and relationships within the ensemble like?*
    - *How do you work as an actor today?*
    - *How do you approach your roles?*
    - *How does it feel to work with the stories of the different roles?*
    - *What is it like to play a leading role versus supporting roles?*

Topic 3: What makes a good day?

- **Can you tell me about a time when you had a good day at work?**
    - *Can you describe the room you were in, what it smelled like, how many people were there, etc.?*
    - *How did it feel?*
    - *What were you thinking in that situation?*
    - *What was it that made that particular day so lovely?*
- **Can you tell me about a time when you had a difficult day at work?**
    - *Can you describe the room you were in, what it smelled like, how many people were there, etc.?*
    - *How did it feel?*
    - *What were you thinking in that situation?*
    - *What was it that made that particular day so bad?*
- **If you were to describe a perfect day at work, what would it look like?**
- **How far away are you from that 'perfect day' today?**

Topic 4: The meaning of an actor's life

- **What do you find rewarding about being an actor?**
- **What do you find tiring about being an actor?**
- **How has this evolved over the course of your career?**
