# Supplementary material for: Hidden struggles: professional Norwegian actors’ experiences with performance anxiety and its consequences in their daily work
Source: Front Psychol. 2026 Jul 10;17:1803171. doi: 10.3389/fpsyg.2026.1803171 (PMC13397583; doi:10.3389/fpsyg.2026.1803171)
Supplement: Supplementary file 2 [file Data_Sheet_2.PDF]

Appendix 2

Example of thematic narrative analysis

Story 1: 'No one wants to hire a mad actor'

Although I knew that being an actor was what I wanted to do for the rest of my life, I`ve had this anxiety inside me, which started in theatre school. I had never had any problems with my mental health in my youth, but through the years in theatre school, I experienced some episodes of problems with heavy breathing. The years in school were tough, and several students decided to quit because they realised that this life wasn't for them. It was like a constant pressure that pushed me forward and forced me to develop my skills and talent, but also pressed the air out of my lungs and left me gasping.

This story starts on a regular afternoon in the autumn. I am in a Scandinavian city, and through a series of lucky coincidences, I have been given a big chance in a great show on one of the biggest stages in the country. The play runs its course, and I feel like the luckiest person in the world. Here I am, on this big stage standing alongside these famous actors I have only dreamed of working with!

I am standing still on the stage, and suddenly my arm starts to shake. It is the twentieth-something show, and I have felt fine until this day. I just can't stand still. It is quite easy to spot the only actor moving when everybody is supposed to stand still. 'Oh no', I say to myself. 'This is crazy! How can I be an actor if I can`t stand still on stage?' The problem returns every night. In the same scene, I start shaking when I am supposed to stand still.

Then, I am in a new city, in a big new production, and I feel fine. I feel fine during the rehearsals, I feel fine during the first live shows. But when I get to the twentieth-something show, the problem is back: I can't stand still. 'This is it', I think to myself. 'No one wants to hire an actor who can't stand still'. The problem continues, and the minute I walk on stage it is getting hard to breathe. I have developed routines I just have to do to prevent me from not shaking on stage. Now it is not just on stage that I have problems, but my private life has also been infected by this fear of losing control. 'I'm going crazy', I keep saying to myself. 'No one wants to hire a mad actor!'

At last, I think I am having a heart attack on stage. The pain in my chest is overwhelming, and I can barely stand on my feet. It is hard to breathe, and again I find myself gasping for air.

The next day I go to the doctor, who says, 'You are not dying. But you have all the signs of anxiety and OCD [obsessive-compulsive disorder].

Step 1: Holistic reading of the narrative

The narrator is an actor describing how the school's elimination system stirred up a sense of unease within him, which came to a head when his career peaked early on. Through contacts and other acting gigs, he landed a role in a production at a major theatre, followed shortly afterwards by another role at a different major theatre, which made him feel the pressure to perform, if only to justify the fact that he'd been given the chance. He thinks it would have been easier if he'd worked his way up the ladder more slowly and, for example, gotten a job at a smaller theatre first. He believes the rush into the new jobs led to him having trouble standing still during performances but not during the rehearsal period. And he developed obsessive thoughts that if he moved now, he wouldn't get any more work, and so he had to move.

He describes the industry as 'wishy-washy', as one in which you get jobs largely through contacts and chance and by being in the right place at the right time. That perception makes for a rather uncertain daily life, and he reflects a great deal on what it's like to be in that situation and what it does to one's mental health. He feels that you always have to be thinking about the next job, which makes it difficult to talk about difficult things. It meant he didn't go to anyone at the theatre or in his private life for fear of being called 'crazy' and difficult to work with.

The turning point of the story is when the participant thought he'd had a heart attack, which led him to see his general practitioner, who said he was showing signs of panic attacks. That news led to him being referred to a psychologist, but as the waiting list was so long and he was not acutely ill, he went to a private psychologist instead, whom he paid for out of his own pocket. Then, at the general practitioner's, the doctor did not entirely believe all the symptoms of mental ill health that he described because they did not quite fit the textbook definition due to becoming apparent on stage only.

Step 2: Narrative meaning units

- I haven’t really struggled with depression, but I have struggled with a lot of anxiety—a great deal of anxiety—which, in a way, started back when I was training as an actor.
- I spent a year freelancing after graduation when there wasn’t much work, and suddenly I was given a big opportunity at a repertory theatre.
- [It was] a brilliant opportunity, but it meant that you were constantly—you’d bump into, like, big-name actors in the canteen every day, and it all just became a bit absurd.
- And then, um, at the twentieth performance, I suddenly started having trouble standing still on stage.
- And you get absolutely terrified that others will see it. That the other actors will think, ‘Blimey, he’s lost it’.
- Because I’m afraid I won't get any more work. The physiological problems evolve from shaking and freezing to heavy breathing on stage.
- Because I was so scared of being ... I mean, I was so devastated by it, by that feeling that you’re being written off as a mad person, you know.
- ‘But you have them all,’ the doctor said. ‘You have all the symptoms of a panic attack’.

Step 3: Recurring themes across the stories (some units have been omitted to prevent identification)

Theme 1: Big shoes to fill – Precarious professional legitimacy and escalating internalised standards

- I spent a year freelancing after graduation when there wasn’t much work, and suddenly I was given a big opportunity at a repertory theatre. (Jens)
- [It was] a brilliant opportunity, but it meant that you were constantly—you’d bump into, like, big-name actors in the canteen every day, and it all just became a bit absurd. (Jens)
- I was completely overwhelmed by being allowed to work on something I loved. And a bit unsure whether I actually had the right to do it. (Nina)
- And I wanted to hide this from everyone, because, I mean, I’m known for being bloody good with lines. (Janne)

Step 4: A narrative exploration of the topic

The analysis shows an ambivalence between acknowledgement and doubts about legitimacy. Being offered an acting job generates strong feelings of happiness and humility of being allowed to work with acting. At the same time, it also establishes an insecurity of one’s talent and an eagerness to prove one’s worth of the job. Having a feeling of being lucky or working in high-profile productions seems to intensify the ambivalence.

Putting those feelings into words seems to mark a turning point and builds resilience. Sharing such feelings is difficult due to the fear of getting a bad reputation and losing future job opportunities.
