# Supplementary material for: Hidden struggles: professional Norwegian actors’ experiences with performance anxiety and its consequences in their daily work
Source: Front Psychol. 2026 Jul 10;17:1803171. doi: 10.3389/fpsyg.2026.1803171 (PMC13397583; doi:10.3389/fpsyg.2026.1803171)
Supplement: Supplementary file 3 [file Data_Sheet_3.pdf]

Appendix 3

Reflexive Thematic Analysis for Theme 1

Reflexive Thematic Analysis for Theme 1		
**Meaning unit**	**Subtheme**	**Theme**
I've felt it before … that people around me say, 'Isn't he going to quit soon?' or 'Isn't it time he looked in the mirror and saw how bad he is?' Well, that doesn't quite match the reactions I've gotten to things I've done, but still, there's this nagging feeling that I'm not good enough … that I myself should at least make the choice to start something else. (Kristian)	**Fear of others' judgement**	**Big shoes to fill – Precarious professional legitimacy and escalating internalised standards**
You're terrified that others will see it, that other actors will think, 'Oh, he's lost it'. (Jens)		
Yeah, my nerves are getting worse and worse. I reckon it's down to age. I thought maybe some people become more like, 'Nah, I don't give a damn what people think of me' as they get older. … But I don't feel it's been quite like that for me. I feel it's perhaps been a bit the other way round, that you become a bit more self-conscious about things. (Katrine)		
Well, and when I finally got that job in [theatre], it was because someone else in the class had tipped me off that he'd turned it down. So, I seized the opportunity and sent a message to [theatre]. So that's how it went. It felt like a second choice. (Per)	**Getting the job "by accident"**	
Suddenly, there was this actor in the theatre who was actually supposed to be in this production but couldn't make it, so the director asked, 'Do you have anyone you'd recommend?' 'Yes, I recommend Jens.' And so it worked out, and it turned out that the director had seen me in a theatre group the year before. That's just how coincidental it is. (Jens)		
I was completely overwhelmed by being allowed to work on something I loved. And a bit unsure whether I actually had the right to do it. (Nina)		
[It was] a brilliant opportunity, but it meant that you were constantly—you'd bump into, like, big-name actors in the canteen every day, and it all just became a bit absurd. (Jens)		

We were sort of 10 people selected from a pool of so-and-so many. That was basically the recipe for getting stressed out then, because of the fear of not being able to fill that place. I think there were several of us who went around with the feeling that 'They've made a mistake here; it wasn't me they were supposed to have. They'll surely soon realise they've made a mistake. (Per)	**Not deserving the job**	
I kept thinking, 'Here are all the more experienced ones'. So the likelihood of me getting in meant I might have to work even harder because I felt I had something to prove back then, that I was progressing faster because I'm not, I wasn't as good as the others when we started. It was definitely in my head. It wasn't something the teachers ever said. (Ida)		

***Note*. Some meaning units have been excluded from the table to protect the participants' anonymity.**

Reflexive Thematic Analysis for Theme 2

Reflexive Thematic Analysis for Theme 2		
**Meaning unit**	**Subtheme**	**Theme**
I had spent the whole weekend learning the new songs, and one of them was a solo song. Then I arrived on Monday, and the first thing I know is that the solo song has been cancelled. ... And so, well, I couldn't do it; I couldn't do the songs I've practised. ... Everything was in place for me to perform, and then I couldn't do it .... So when the day ended ... I noticed that the new songs that we learned that day didn't stick, because my head had started thinking, 'Why did they cut it [the solo song]?'—because I can't sing'. (Veronica)	**Lack of feedback**	**Small sparks can cause big fires – Trigger events that destabilise perceived competence and precipitate spirals of anxiety**
You can't see yourself from the outside, so you rely on feedback to hear that, well, you were sort of what they wanted, and when you don't get that, you have to sort of interpret signals and try to figure out if you did the right thing. (Andy)		
Even after leaving [acting school], I still hadn't managed to learn how to use my creativity. I remember so clearly the last 6 months [at acting school] when one director was just like, 'You have to improvise! Improvise!' And no one dared to improvise anymore because, well, every time we tried something, it was wrong. (Nina)	**Never good enough**	
And then I felt a bit of a threat there, like, 'Well, if you can't do this, then we'll just have to choose the other person'. And I found that difficult, when someone says something like that. *[Pause]*. That sort of psychological pressure doesn't work on me. It actually just made me feel more scared for next time. I was scared I wouldn't remember anything. It triggered a fear response in me then. (Kari)		
I started getting anxiety that got so bad I couldn't stand still in a scene as simple as that. Often in the theatre world—and this is especially true in musicals—you		

suddenly 'freeze'; you just 'freeze' right in the middle of a scene, whilst something important is happening under a spotlight or something, you know. And that's when I suddenly started getting this really bad shaking just from standing still. (Jens)	**Small events with big impacts**	
And then we turned the camera round, and they filmed us, there were several others on the other side there. And then suddenly I stumbled just a tiny bit. And then I got this feeling of 'Oh! What if, what if this is a possibility? What if this could happen?' And then that thought started spinning until I managed to twist myself into a kind of performance anxiety hell. (Janne)		

***Note.* Some meaning units have been excluded from the table to protect the participants' anonymity.**
